# Proteomic subtyping of Alzheimer's disease CSF links blood‐brain barrier dysfunction to reduced levels of tau and synaptic biomarkers

**DOI:** 10.1002/alz70856_107354

**Published:** 2026-01-09

**Authors:** Madison Bangs, Joshna Dharmendrabhai Gadhavi, Emma Kathleen Carter, Lingyan Ping, Duc M Duong, Eric B. Dammer, Anantharaman Shantaraman, Caroline M Watson, Edward J Fox, Erik C.B. Johnson, James J. Lah, Allan I. Levey, Nicholas T Seyfried

**Affiliations:** ^1^ Emory University, Atlanta, GA, USA; ^2^ Emory University School of Medicine, Atlanta, GA, USA; ^3^ Goizueta Alzheimer's Disease Research Center, Emory University, Atlanta, GA, USA; ^4^ Emory University Center for Neurodegenerative Disease, Atlanta, GA, USA; ^5^ Department of Neurology, Emory University School of Medicine, Atlanta, GA, USA

## Abstract

**Background:**

Neuropathological changes in Alzheimer's disease (AD) begin decades before cognitive symptoms appear, highlighting the need for early detection to enable prevention and treatment. The ATN framework (amyloid [A], tau [T], and neurodegeneration [N]) is widely used for classifying AD, but biomarker progression often varies due to factors such as genetics, sex, race, and environment. AD is also characterized by significant heterogeneity, with comorbidities like cerebrovascular disease and Lewy body disease complicating diagnosis and treatment. Molecular subtyping has emerged as a promising approach to address this complexity, yet its application across diverse populations remains limited.

**Method:**

Following tandem mass tag labeling, a network‐based analysis was applied to the CSF proteome (*n* = 2,067 proteins) from 483 samples (245 control, and 238 AD) that included 130 samples from African Americans, to identify molecular subtypes of AD. Proteomic data were organized into 10 network modules associated with molecular pathways, functions, and brain cell types. Using clustering techniques, we identified six molecular subtypes comprised of both AD and control samples, and examined their relationships with age, sex, race, and established AD biomarkers (Aβ, tau, pTau). Validation was performed with independent proteomic datasets, and plasma‐CSF dilution experiments were conducted to explore the role of proteolytic enzymes in blood‐brain barrier (BBB) dysfunction on CSF tau and synaptic protein levels.

**Result:**

We identified six CSF proteomic subtypes, which largely aligned with previously described categories defined by proteins enriched in neuronal hyperplasticity, immune activation and BBB integrity. African Americans and males were disproportionately represented in the BBB integrity subtype, which was characterized by low CSF tau, high CSF/serum albumin ratios, and reduced synaptic protein levels. This subtype was enriched in proteolytic enzymes such as thrombin, plasminogen, and matrix metalloproteases, which can cleave tau. *Ex vivo* plasma‐CSF dilution experiments confirmed that increasing plasma levels reduced CSF tau and synaptic proteins, likely due to proteolytic activity.

**Conclusion:**

This study highlights network‐based approaches in identifying molecular subtypes of AD that account for clinical and pathological heterogeneity. The BBB integrity subtype highlights how biological traits such as concomitant comorbidities influence CSF biomarkers, providing insights into disease mechanisms and opportunities for diversity‐informed diagnostics and therapies.

